# Validation of a laser projection platform for the preparation of surgical patches used in paediatric cardiac surgery

**DOI:** 10.1093/icvts/ivad129

**Published:** 2023-08-09

**Authors:** Tiffany Saunders, Dominic Recco, Nicholas Kneier, Shannen Kizilski, Peter Hammer, David Hoganson

**Affiliations:** Department of Biomedical Engineering, Worcester Polytechnic Institute, Worcester, MA, USA; Department of Cardiac Surgery, Boston Children’s Hospital, Harvard Medical School, Boston, MA, USA; Department of Cardiac Surgery, Boston Children’s Hospital, Harvard Medical School, Boston, MA, USA; Department of Cardiac Surgery, Boston Children’s Hospital, Harvard Medical School, Boston, MA, USA; Department of Cardiac Surgery, Boston Children’s Hospital, Harvard Medical School, Boston, MA, USA; Department of Cardiac Surgery, Boston Children’s Hospital, Harvard Medical School, Boston, MA, USA

**Keywords:** Congenital heart disease, Cardiothoracic surgery, Surgical planning, Medical imaging, Personalized precision medicine

## Abstract

**OBJECTIVES:**

Reconstruction of cardiovascular anatomy with patch material is integral to the repair of congenital heart disease. We present validation of a laser projection platform for the preparation of surgical patches as a proof-of-concept for intraoperative use in patient-specific planning of paediatric cardiac surgery reconstructions.

**METHODS:**

The MicroLASERGUIDE, a compact laser projection system that displays computer-aided designs onto 2D/3D surfaces, serves as an alternative to physical templates. A non-inferiority comparison of dimensional measurements was conducted between laser projection (‘laser’) and OZAKI AVNeo Template (‘template’) methods in creation of 51 (each group) size 13 valve leaflets from unfixed bovine pericardium. A digital version of the OZAKI AVNeo Template dimensions served as control. Feasibility testing was performed with other common patch materials (fixed bovine pericardium, PTFE and porcine main pulmonary artery as a substitute for pulmonary homograft) and sizes (13, 23) (*n* = 3 each group).

**RESULTS:**

Compared to control (height 21.5, length 21.0 mm), template height and length were smaller (height and length differences of −0.3 [−0.5 to 0.0] and −0.4 [−0.8 to −0.1] mm, *P* < 0.01 each); whereas, both laser height and length were relatively similar (height and length differences of height 0.0 [−0.2 to 0.2], *P* = 0.804, and 0.2 [−0.1 to 0.4] mm, *P* = 0.029). Template percent error for height and length was −1.5 (−2.3 to 0.0)% and −1.9 (−3.7 to −0.6)% vs 0.2 (−1.0 to 1.1)% and 1.0 (−0.5 to 1.8)% for the laser. Similar results were found with other materials and sizes. Overall, laser sample dimensions differed by a maximum of 5% (∼1 mm) from the control.

**CONCLUSIONS:**

The laser projection platform has demonstrated promise as an alternative methodology for the preparation of surgical patches for use in cardiac surgery. This technology has potential to revolutionize preoperative surgical planning for numerous congenital anomalies that require patient-specific patch-augmented repair.

**Figure:**
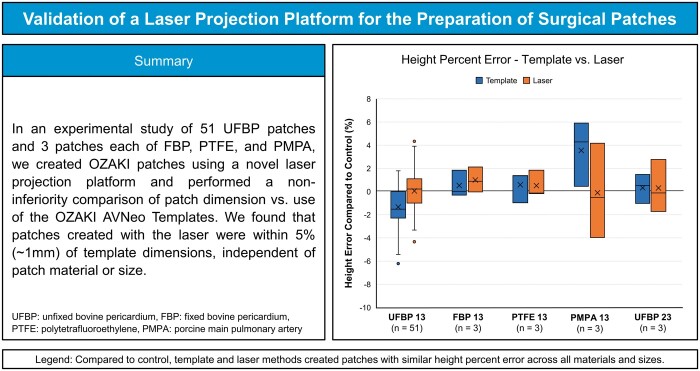

## INTRODUCTION

Reconstruction of cardiovascular anatomy with patch material is an integral component of surgical repair for congenital anomalies including aortic valve (AV) disease, Tetralogy of Fallot, atrioventricular septal defects, total anomalous pulmonary venous return, intracardiac baffles and hypoplastic or interrupted aortic arch [1–6]. Despite advances in surgical techniques and technology, much of the patch shape and size is still determined intraoperatively and guided primarily by surgeon experience and visual estimates. Often, patches need to be resized multiple times prior to implantation to achieve the desired reconstructed anatomy. Surgical outcomes continue to have substantial geometric variability, requiring reoperation for consequences of under- or oversizing the patch [7–13]. To facilitate operational accuracy and improve postoperative outcomes, patient-specific 3D computational models developed from preoperative imaging have become integrated within the surgical planning process at several paediatric heart centres [14–17]. However, translation of the modelled anatomy and patch repair into the operating room (OR) continues to be a limiting factor in the workflow.

Advances in technology have been leveraged in other arenas to accurately and consistently translate complex geometric designs for manual fabrication. Laser projector systems are currently being used in the aerospace industry to replace physical templates and cumbersome manual measurement procedures to provide true-to-scale alignment and positioning for manufacturing and assembly [18]. We hypothesized that laser projection would be an efficient and accurate means to display 3D-model-generated patch templates within the OR for use by the operating surgeon. A laser-based system can serve as an alternative to physical templates, which may be costly and/or require sterilization between uses. Furthermore, patient-specific patch planning implies that each physical template would only be useful for the index patient, which may be impractical given the long lead times and substantial cost of 3D-printed or machined patient-specific templates. Prior to adoption of an existing device into a different field such as healthcare, validation of its capabilities in an intended specialty (cardiac surgery) and use (patch planning) was deemed necessary. Therefore, we sought to evaluate our proposed methodology against a standardized surgical procedure, AV neo-cuspidation using the Ozaki technique, prior to intraoperative implementation.

The Ozaki surgical technique for AV reconstruction has been previously described [5]. Briefly, harvested autologous pericardium is placed on a surface under tension and ‘fixed’ with 0.625% glutaraldehyde for use as the new AV leaflets (i.e. cusps). Glutaraldehyde-fixed tissues are used for their increased tensile strength and better handling capabilities. The native AV leaflets are excised. Intercommissural distances are measured with the OZAKI AVNeo Sizer System (JOMDD, Tokyo, Japan). Using the corresponding OZAKI AVNeo Template size (range from 13 to 31 mm), the leaflet material is marked along the outer periphery. The neo-cusps are cut out and sewn into the aortic root. Millimetre-level accuracy is required to recreate normal AV geometry and to prevent poor surgical outcomes (e.g. residual regurgitation/stenosis, reintervention). Given the procedure’s demand for reproducibility and precision, the Ozaki technique, specifically the template methodology, served as a control for our experiment. Herein, we report a validation of the proposed laser projection platform in the preparation of surgical patches for use in cardiac surgery.

## MATERIALS AND METHODS

### Ethics statement

*In vitro* studies performed with exemption by BCH IRB.

### Laser projector technology

**Figure 1:**
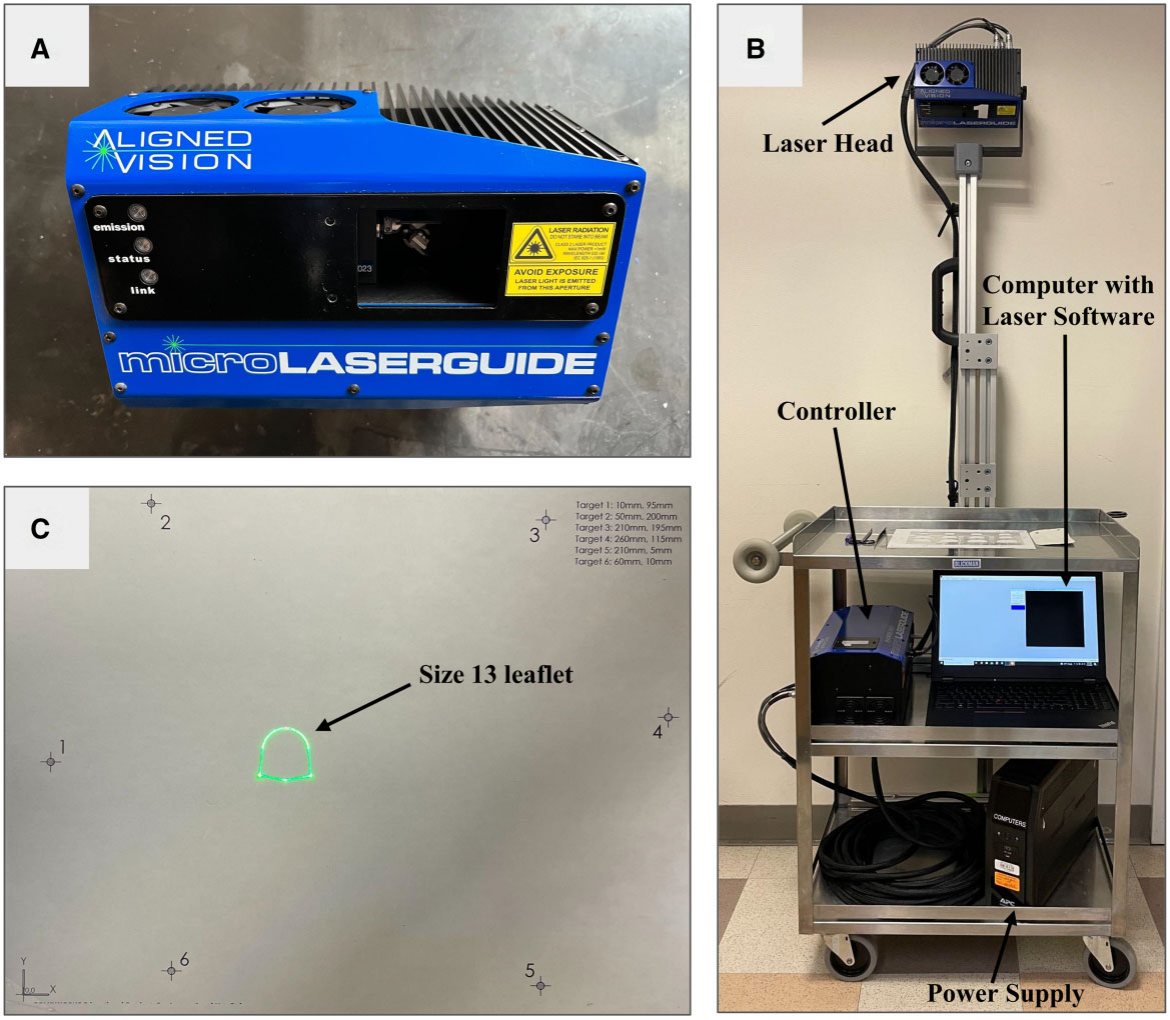
(**A**) MicroLASERGUIDE laser head. (**B**) Preliminary design of the mobile laser system for use in the operating room. The MicroLASERGUIDE laser head is mounted on 30 mm × 30 mm aluminium extrusion attached to a stainless steel cart. After sterile draping, the desired shape is projected onto the top surface where the surgeon prepares the patch for use in repair. The laser controller and computer with LASERGUIDE software are located on the cart underneath the working surface. (**C**) Laser registration targets (numbered), 6 in total, used for creating the transform to properly project onto the desired surface. Laser projection of a size 13 OZAKI AVNeo Template (arrow) shown in the centre of the calibrated surface.

The MicroLASERGUIDE (Aligned Vision, Chelmsford, MA) system is a compact mobile laser projection system used in aerospace, automotive and wind energy industries to provide manufacturing templates and quality control capabilities. The system consists of 3 main components: the laser head (MicroLASERGUIDE), the controller and the computer with LASERGUIDE software (Fig. 1A and B). The laser projects computer-aided designs (CAD) onto 2D/3D surfaces using a Class 2 laser (no safety eyewear required), moving the laser spot rapidly through the prescribed sequence of coordinates. This rapid movement allows for the appearance of a singular outline or shape (Fig. 1C). The laser unit weighs 8 pounds and measures 8.5 × 4.5 × 5.5 in., which allows for easy mounting and relocation (Fig. 1A and B). The CAD model of the desired shape is exported as a .TXT file formatted to be read by the LASERGUIDE software. Laser projection files are uploaded to the system through a computer communicating with the controller. The laser projection file consists of laser registration targets, used to calibrate the projection to the desired surface, and a list of projection coordinates for the design of interest. The user steers the laser via the software to each of the 6 registration markers on the desired surface to align and orient the projection field. After proper calibration, the uploaded design is displayed (Fig. 1C). The system can project onto 3D curved surfaces with an accuracy of 0.015″ (0.38 mm) from 3 to 15 ft away.

### Experimental groups

This study was designed to evaluate the accuracy of laser-projected patch plans as an alternative to the standard physical template method of translating patch design to sterile patch materials. To evaluate and validate the performance of the laser projection system for patch planning, AV leaflets generated by laser projection (henceforth referred to as the ‘laser’ method) and the OZAKI AVNeo Template (henceforth referred to as the ‘template’ method) were compared. The laser-projected leaflet design was sized according to a CAD file developed from the measurements outlined in the OZAKI Template patent (‘Template for forming valve leaflet’) (henceforth referred to as the ‘control’) [19]. The CAD model consisted of a dimensioned sketch prepared by an engineer (N.K.) using the line and arc elements in SolidWorks (Dassault Systèmes SolidWorks Corporation, Waltham, MA). The control was dimensionally equivalent to the physical template dimensions. To expand the generalizability of the results, 4 different patch/leaflet materials were used: unfixed bovine pericardium (UFBP) and fixed bovine pericardium (FBP) as substitutes for unfixed/fixed autologous pericardium, polytetrafluoroethylene (PTFE) and porcine main pulmonary artery (PMPA) as a substitute for thick patch pulmonary homograft. Additionally, both Ozaki template sizes 13 and 23 were studied for UFBP. In total, 5 experimental groups, (i) size 13 UFBP, (ii) size 13 FBP, (iii) size 13 PTFE, (iv) size 13 PMPA and (v) size 23 UFBP (Table 1), were tested to assess the accuracy and precision of the laser compared to the template method across different patch/leaflet materials used in cardiac surgery applications.

**Table 1:** Experimental groups for each method

Leaflet material	Ozaki AVNeo Template size	Sample size per method
Unfixed bovine pericardium (UFBP)	13	51
Fixed bovine pericardium (FBP)	13	3
Polytetrafluoroethylene (PTFE)	13	3
Porcine main pulmonary artery (PMPA)	13	3
Unfixed bovine pericardium (UFBP)	23	3

### Validation study design

**Figure 2:**
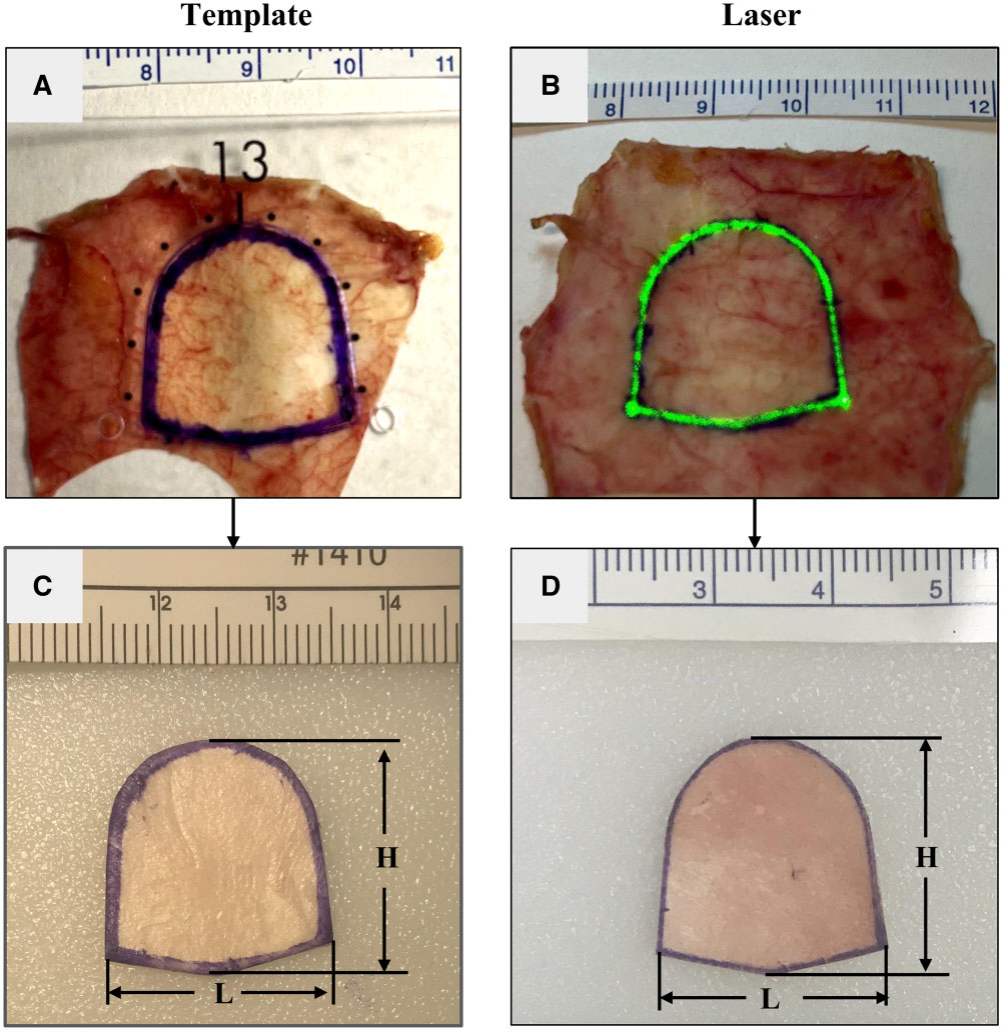
(**A**) Representative photo of the template method. Size 13 leaflet traced on an unfixed bovine pericardium (UFBP) sample placed on a surgical gown pass using the Ozaki AVNeo Template (clear plastic) by marking along the outer periphery. (**B**) Representative photo of the laser method. Size 13 leaflet traced on a UFBP sample placed on a surgical gown pass card using laser projection (marked along the middle of the laser-projected line). (**C**) Representative photo of a cut-out size 13 UFBP leaflet using the template method. Note that the sample was cut on the outside portion of the marked line (∼25% of the thickness from the outer edge). (**D**) Representative photo of a cut-out size 13 UFBP leaflet using the laser method. Note that the sample was cut in the middle of the marked line. The dimensional lines in (**C**) and (**D**) correspond to the measurements (‘H’ for height, ‘L’ for length) recorded using ImageJ and a ruler for scaling. Rulers in (**A**)–(**D**) have centimetre increments numbered.

Leaflets were created in a way that emulated current OR practices at our institution. The leaflet material was placed on a surgical gown pass card and the OZAKI AVNeo Template was used to trace a leaflet of desired size using a VISCOT skin marker 1450XL (Viscot Medical, LLC, East Hanover, NJ) (Fig. 2A). The leaflet was then cut out with a pair of FEHLING BOT-1 scissors (Fehling Surgical Instruments, Inc., Kennesaw, GA) along with the underlying pass card. The template samples were cut on the outside portion of the marked line (∼25% of the thickness from the outer edge) as the leaflet is traced inside of the appropriately sized template with some ink bleed over (Fig. 2A and C). For the laser samples, the material was cut on the centre of the marked line, as the leaflet is drawn directly overlying the laser-projected design (Fig. 2B and D). Unlike most available patch materials (e.g. PTFE and thick pulmonary homograft), autologous pericardium requires an additional step prior to tracing. Per our institutional protocol, autologous pericardium is placed in a 0.625% glutaraldehyde bath for 5 min and subsequently rinsed twice with normal saline for 3 min. Of note, our surgeons find it helpful to affix the edges of the pericardium to the pass card with surgical clips to minimize curling during the fixation process. To mimic this protocol, FBP was prepared, marked and cut in a similar fashion. These procedural steps were followed as detailed to complete all validation experiments. Photos of the cut-out leaflets alongside a ruler were analysed with ImageJ (Oracle, Austin, Texas), and height and length measurements were recorded (Fig. 2C and D). For consistency, 1 surgeon (Dominic Recco) performed all of the leaflet tracing and cutting and 1 engineer (Tiffany Saunders) performed all of the leaflet measurements.

### Statistical analysis

Descriptive analyses were performed within each method (i.e. laser and template) and between each method and the control dimensions. Within-group central tendency and variation of leaflet measurements are presented as mean ± standard deviation (SD). Method comparisons to the control dimensions by difference (i.e. measured dimension − control dimension) and percent error (PE) [i.e. (measured dimension − control dimension)/control dimension × 100] are presented as median (Q1–Q3). Inferential statistical analysis via a non-inferiority comparison was conducted for UFBP, whereas only descriptive statistical analyses were conducted for the remaining materials due to small sample sizes. Deviation between methods was assessed with the unpaired Student’s *t-*test and differences between each method and the control were assessed with a one-sample *t-*test. To achieve adequate statistical power, sample size calculations utilizing a non-inferiority design were performed. The non-inferiority margin was defined based on statistical results from a pilot study (*n* = 9) and clinical judgement. Preliminary testing resulted in a pooled SD of 0.46 mm for height. A preserved fraction of 50% was used, which is common practice in non-inferiority trials [20, 21], resulting in a margin of 0.23 mm or an absolute difference of 11% from the pooled sample mean. Assuming a mean difference of zero (i.e. identical leaflet sizes between the 2 methods), sampling ratio of 1, power of 80% and type I error rate of 5%, a group sample size of 51 was calculated. All analyses were conducted using R version 3.6.3 (R Foundation for Statistical Computing, Vienna, Austria), and *P*-values <0.05 were considered statistically significant.

## RESULTS

### Non-inferiority comparison of size 13 unfixed bovine pericardium

Size 13 leaflet samples of UFBP were created using both the template and laser (*n* = 51 in each group) to statistically compare the 2 methods. The laser-projected CAD file measured 21.5 mm in height and 21.0 mm in length to reflect the OZAKI AVNeo Template patent dimensions for the size 13 leaflet.

#### Template versus control

**Figure 3:**
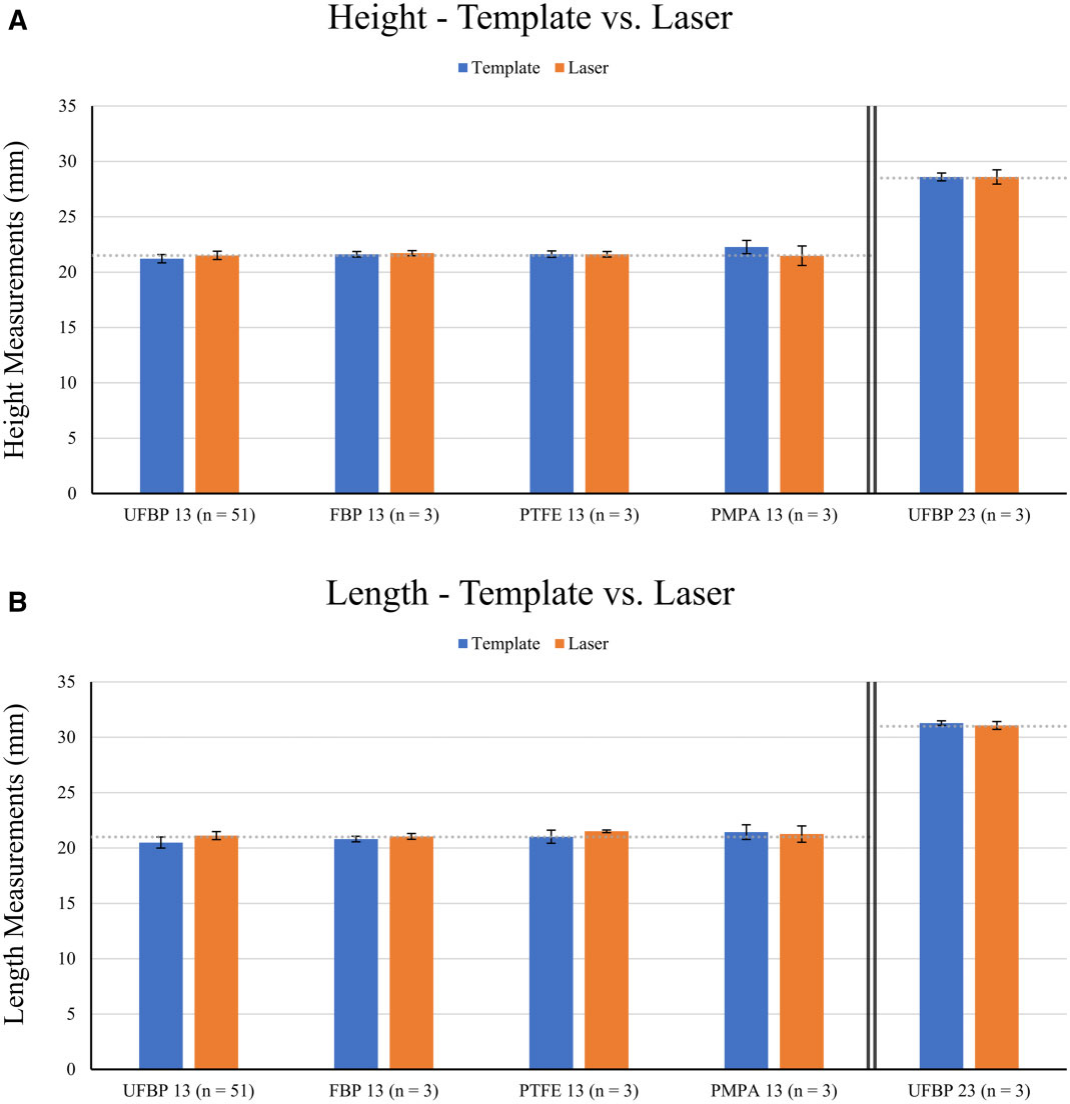
(**A**) Height measurements for the template (left) and laser (right) methods across all experimental groups. (**B**) Length measurements for the template (left) and laser (right) methods across all experimental groups. For both (**A**) and (**B**), the top of the bar represents the average of the measurements, and the error bars represent the standard deviation. OZAKI AVNeo Template sizes 13 and 23 are denoted by ‘13’ and ‘23’ next to each patch material. The dotted line denotes the OZAKI AVNeo Temple patent height and length dimensions for the size 13 (21.5 and 21.0 mm) and 23 (28.5 and 31.0 mm) sizers. FBP: fixed bovine pericardium; PMPA: porcine main pulmonary artery; PTFE: polytetrafluoroethylene; UFBP: unfixed bovine pericardium.

**Figure 4:**
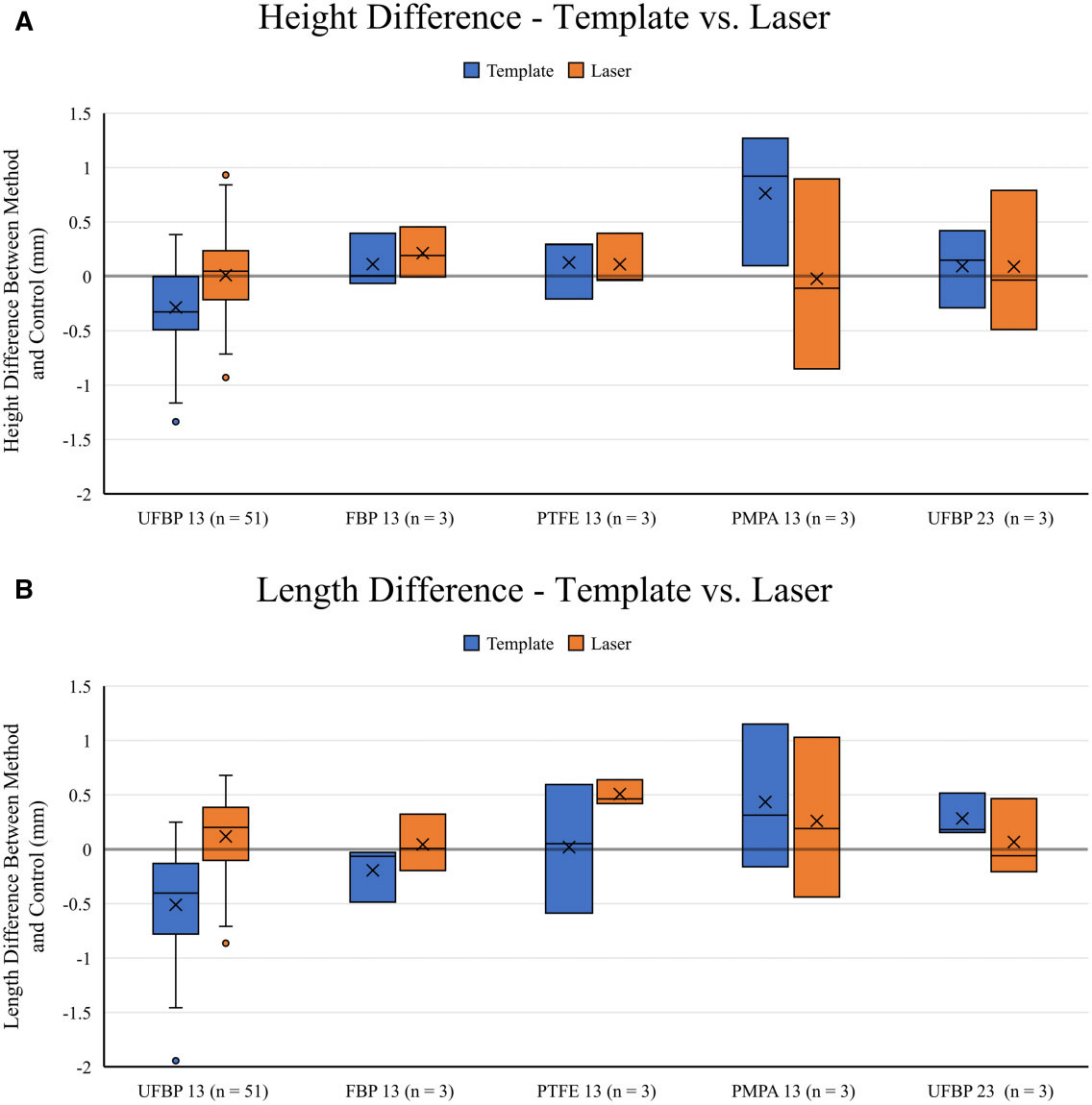
(**A**) Box-and-whisker plots of the height difference (measured height—control height) for the template (left) and laser (right) methods across all experimental groups. (B) Box-and-whisker plots of the length difference (measured length—control length) for the template (blue) and laser (orange) methods across all experimental groups. OZAKI AVNeo Template sizes 13 and 23 are denoted by ‘13’ and ‘23’ next to each patch material. For both (**A**) and (**B**), the abbreviations are the same as in Fig. 3.

**Figure 5:**
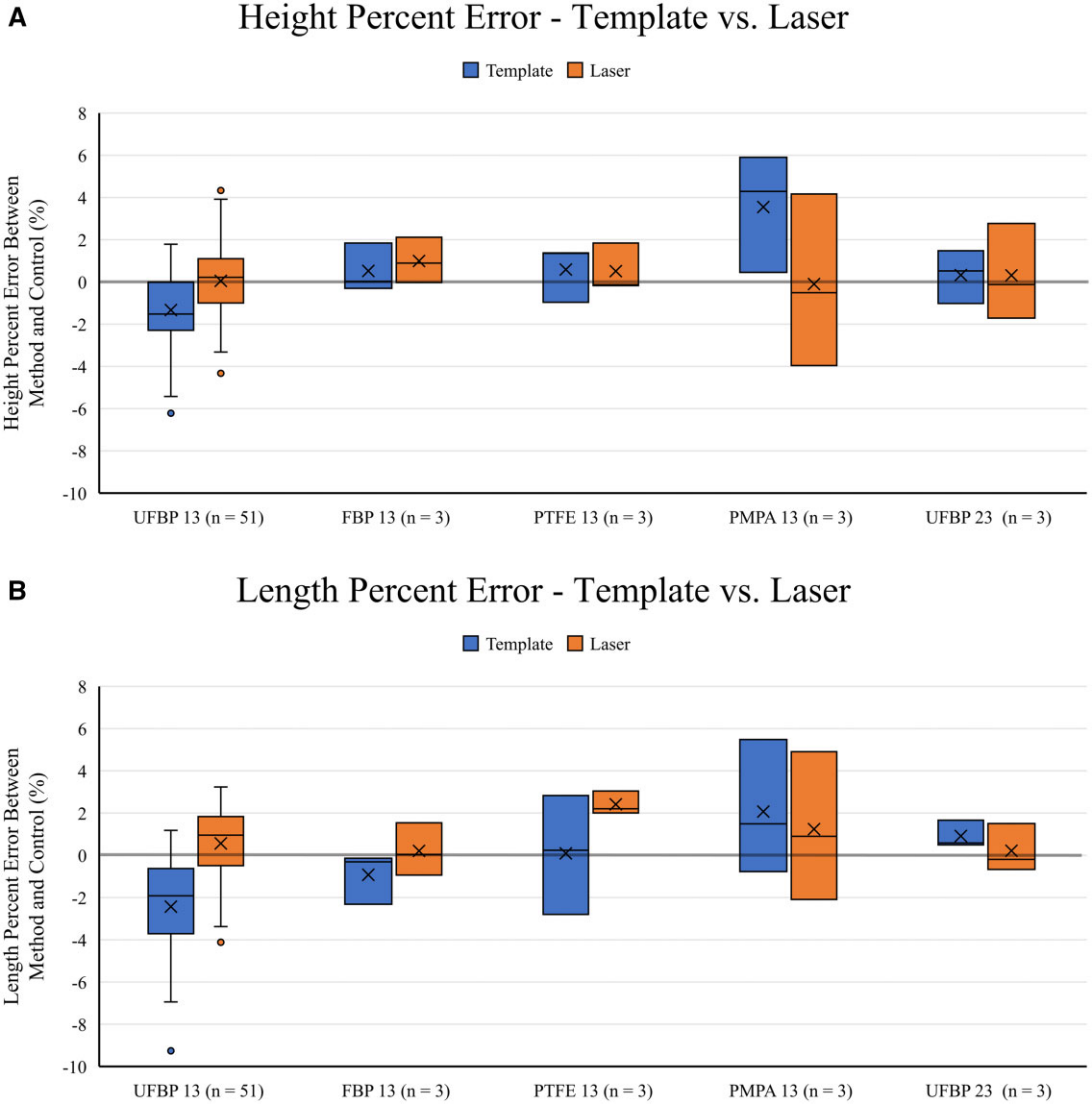
(**A**) Box-and-whisker plots of the height % error [(measured height − control height)/control height] for the template (left) and laser (right) methods across all experimental groups. (**B**) Box-and-whisker plots of the length % error [(measured length − control length)/control length] for the template (blue) and laser (orange) methods across all experimental groups. For both (**A**) and (**B**), the abbreviations are the same as in Fig. 3.

The height and length for template samples were 21.2 ± 0.4 and 20.5 ± 0.5 mm (Fig. 3A and B) with similar measurement variability in both dimensions (SD 0.4 vs 0.5 mm). Both template height and length were statistically smaller than the control (21.2 vs 21.5 and 20.5 vs 21.0 mm). These measurements corresponded to height and length differences of −0.3 (−0.5 to 0.0) and −0.4 (−0.8 to −0.1) mm (*P* < 0.01 for both) (Fig. 4A and B). The template PE for the height and length was −1.5 (−2.3 to 0.0)% and −1.9 (−3.7 to −0.6)% (Fig. 5A and B).

#### Laser versus control

For the UFBP laser samples, the height and length were 21.5 ± 0.4 and 21.1 ± 0.4 mm (Fig. 3A and B) with identical measurement variability in both dimensions (SD 0.4 mm for both). Both laser height and length were relatively similar to the control (21.5 vs 21.5 and 21.1 vs 21.0 mm; height and length differences of 0.0 [−0.2 to 0.2], *P* = 0.804, and 0.2 [−0.1 to 0.4] mm, *P* = 0.029) (Fig. 4A and B). The laser PE for the height and length was 0.2 (−1.0 to 1.1)% and 1.0 (−0.5 to 1.8)% (Fig. 5A and B).

#### Laser versus template

When comparing the 2 methods for UFBP, the laser sample height and length were statistically larger than the respective template sample height and length [height 21.5 vs 21.2 mm (*P* < 0.01), length 21.1 vs 20.5 mm (*P* < 0.01)]. The temple and laser methods demonstrated similar measurement variability in both dimensions (SD 0.4–0.5 mm). The template method resulted in samples with larger height and length differences from the control compared to laser samples (height −0.3 vs 0.0 mm and length −1.9 vs 1.0 mm). As such, the template samples also displayed a larger PE from the control compared to laser samples for both height and length (−1.5 vs 0.2% and −1.9 vs 1.0%).

### Feasibility comparison of other patch materials and sizes

Feasibility testing was conducted with FBP, PTFE and PMPA using the size 13 OZAKI AVNeo Template and with UFBP using the size 23 template (*n* = 3 for each group) to further evaluate the capabilities of the laser projector platform for patch-planning applications across a variety of materials and patch sizes. Due to the small sample sizes used in the feasibility comparison, inferential analyses could not be conducted to determine statistical significance for these experimental groups.

#### Size 13 fixed bovine pericardium

For the size 13 FBP, heights and lengths were 21.6 ± 0.3 and 20.8 ± 0.3 mm for the template vs 21.7 ± 0.2 and 21.0 ± 0.3 mm for the laser, with similar measurement variability between methods (SD 0.3 vs 0.2–0.3 mm) (Fig. 3A and B). The largest absolute difference and PE between the template measurements and the control were 0.5 mm (Fig. 4A and B) and 2.3% (Fig. 5A and B) vs 0.5 mm (Fig. 4A and B) and 2.1% (Fig. 5A and B) for the laser.

#### Size 13 polytetrafluoroethylene

For the size 13 PTFE, heights and lengths were 21.6 ± 0.3 and 21.0 ± 0.6 mm for the template vs 21.6 ± 0.2 and 21.5 ± 0.1 mm for the laser, with similar measurement variability between methods (SD 0.3–0.6 vs 0.1–0.2 mm) (Fig. 3A and B). The largest absolute difference and PE between the template measurements and the control were 0.6 mm (Fig. 4A and B) and 2.8% (Fig. 5A and B) vs 0.6 mm (Fig. 4A and B) and 3.0% (Fig. 5A and B) for the laser.

#### Size 13 porcine main pulmonary artery

For the size 13 PMPA, heights and lengths were 22.3 ± 0.6 and 21.4 ± 0.7 mm for the template vs 21.5 ± 0.9 and 21.3 ± 0.7 mm for the laser, with similar measurement variability between methods (SD 0.6–0.7 vs 0.7–0.9 mm) (Fig. 3A and B). The largest absolute difference and PE between the template measurements and the control were 1.3 mm (Fig. 4A and B) and 5.9% (Fig. 5A and B) vs 1.0 mm (Fig. 4A and B) and 4.9% (Fig. 5A and B) for the laser.

#### Size 13 comparisons using different patch materials

PMPA had the largest overall height and length measurement variability for both methods (template SD 0.6 and 0.7 mm vs laser SD 0.9 and 0.7 mm) (Fig. 3A and B). The large variability was attributed to difficulty with cutting out the thicker tissue, which was evident in both methods. The material with the largest median difference between height and length measurements and control was PMPA [height 0.9 (0.1–1.3) and length 0.3 (−0.2 to 1.2) mm] for the template method. For the laser method, the greatest differences were observed with FBP [height 0.2 (0.0–0.5) mm] and PTFE [length 0.5 (0.4–0.6) mm] (Fig. 4A and B). Similarly, the material that yielded the highest overall median PE was PMPA [height 4.3 (0.5–5.9)%] for the template method and PTFE [length 2.2 (2.0–3.0)%] for the laser method (Fig. 5A and B).

#### Size 23 unfixed bovine pericardium

For the size 23 (height 28.5, length 31.0 mm) UFBP, heights and lengths were 28.6 ± 0.4 and 31.3 ± 0.2 mm for the template vs 28.6 ± 0.6 and 31.1 ± 0.4 mm for the laser, with similar measurement variability between methods (SD 0.2–0.4 vs 0.4–0.6 mm) (Fig. 3A and B). The largest absolute difference and PE between the template measurements and the control were 0.5 mm (Fig. 4A and B) and 1.7% (Fig. 5A and B) vs 0.8 mm (Fig. 4A and B) and 2.8% (Fig. 5A and B) for the laser.

## DISCUSSION

Virtual surgical planning and intraoperative imaging have long been used in the specialties of otolaryngology and neuro-, orthopaedic and craniomaxillofacial surgery [22–25]. However, to the authors’ knowledge, there are no physical or software-based platforms commercially available to facilitate the implementation of preoperatively planned patch designs into the OR. Given its established role in aerospace and engineering applications, and its potential to project detailed designs onto the sterile patch surface, laser projection was deemed a promising technology to address this clinical need. A validation of a laser projection platform in the preparation of surgical patches was performed to understand expected accuracy/precision and refine the tracing techniques relative to the projected laser image prior to clinical application. Our study demonstrated clinically acceptable accuracy and precision for AV neo-cuspidization leaflet design using the laser compared to the template method and control. Through a non-inferiority comparison using UFBP, the laser resulted in similar maximum measurement variability of ∼1 mm, and smaller maximum absolute difference (0.9 vs 1.9 mm) and maximum absolute PE (4.3% vs 9.3%), when compared to the template method across all height and length measurements. Specifically, rather than a limitation of the technology, human error was thought to be a significant contributor of measurement variability. Based on our results with both methods, cutting of the patch appeared to be a main aspect in the workflow that introduced variability, particularly when considering thicker, harder-to-cut materials (i.e. PMPA) compared to thinner, more uniform samples (i.e. PTFE). Furthermore, the UFBP laser samples were determined to be statistically different from the template samples and more representative of the true CAD dimensions. In feasibility testing with other patch materials and sizes, the laser resulted in a slightly larger maximum measurement variability, and slightly smaller maximum absolute difference and maximum absolute PE, when compared to the template method across all height and length measurements and materials. Due to small sample sizes, comparisons of results obtained under feasibility testing were conducted using descriptive statistics and may not reflect significant relationships between the other materials (e.g. FBP, PTFE and PMPA) using the 2 methods. Overall, these data validate the performance of the laser system as a means for projection of patch designs in the OR. Overall, the surgeon can be confident that patches created using the laser display for use in cardiac surgery will be within 5% (∼1 mm) of the targeted dimensions, independent of the patch material or size.

Utilization of the laser projection system for the preparation of cardiovascular surgical patches has numerous potential applications in the field of paediatric heart surgery. For example, a prospective, patient-specific patch-planning workflow is currently under development at our institution to accurately predict patch dimensions required to achieve target aortic size and geometry during initial aortic arch reconstruction with patch augmentation. Within this workflow, reconstructive patches are designed based on the pathologic preoperative anatomy transcribed into 3D computational models and the targeted postoperative aortic dimensional outcomes. Our group has analysed aortic growth after patch-augmented arch repair to identify targeted reconstructed arch size that results in optimal long-term outcomes. This information will serve as input to generate a 3D patch based on existing native anatomy. The 3D patch configuration created from the model is transformed into a 2D geometry to allow for intraoperative use. The finalized 2D patch template is annotated with markers to assist in proper orientation and uploaded to the MicroLASERGUIDE projector software. The patient-specific patch design will be displayed on a specialized mobile cart platform in the OR (Fig. 1a) to allow the surgeon to trace the design onto the patch material for use in arch reconstruction. Although several components of the described methodology are well underway, further research into validating and standardizing this workflow must be conducted prior to clinical application. Moreover, complexity of the proposed algorithm will vary depending on the patient’s anatomy, pathophysiology and intended procedure. Aortic reconstruction with patch augmentation is intricate, demanding millimetre-level precision and an understanding of complex geometry, at times requiring multiple steps of trimming the patch to achieve the desired aortic shape and size. The consequences of inadequate reconstruction can be devastating and associated with lifelong elevated disease risk. A similar workflow is being developed for pulmonary artery patch reconstruction and intraoperative patches including left ventricle to aorta baffles as part of biventricular repair and complete AV canal defect repair. A prospective, patch-planning workflow that incorporates laser projection for use in this setting and in a broad array of other cardiovascular applications may mitigate uncertainty in patch sizing and, ultimately, improve patient outcomes.

Translation of this technology into the OR environment is not without monetary and institutional barriers. The total cost of the laser platform is roughly $50k with minimal maintenance expenses after initial purchase. Per our institutional records, disposable physical templates cost roughly $2–4k per unit. The total hospital expense between the 2 methods breaks even at ∼15 surgical cases, thus favouring long-term use of the laser system. The laser system also has the capability of facilitating both template- and non-template-based operations, increasing its value further. Additionally, the projection source is classified as a Class II laser, similar to neurosurgical units, and, inasmuch, did not require special safety equipment. The mounting cart was inspected and approved by the biomedical engineering department. From a personnel perspective, the platform currently requires an engineer to set up the system and display the desired patch design. However, after the patch geometry is designed, the projection process requires <10 min. On the surface, these barriers may seem to outweigh the benefits of traditional templates and ad hoc patch sizing. However, we aim to eliminate the geometric variability in reconstructed anatomy that contributes to negative long-term patient outcomes. The laser platform provides the ability to display custom, patient-specific patches intraoperatively for a diverse range of cardiovascular applications. With this capability, we are developing a preoperative patch-planning workflow that enables surgeons to achieve precise geometric repairs with patient-specific patch augmentation, streamlining the surgical procedure and facilitating normalization of outcomes across centres.

### Limitations

Due to cost and limited supply, typical commercially available patch materials were substituted to perform the validation and feasibility experiments. Bovine pericardium was used instead of autologous pericardium and porcine pulmonary artery in place of thick patch pulmonary homograft. However, respectively, these materials have similar characteristics including mechanical properties, thickness and surface characteristics. Additionally, the non-inferiority comparison was conducted using UFBP due to limited availability of glutaraldehyde at the time of testing; FBP is used more often in paediatric heart surgery and may have been of more clinical interest. The authors do not believe FBP would have displayed different results as the 2 materials demonstrate similar thickness and pliability when placed on the pass card. Another limitation is the lack of standardization with drying time. When each sample was removed from saline storage and placed on the surgical gown card, it was allowed to dry for an unspecified time to facilitate easier marking. However, shrinkage may have occurred if the sample were allowed to dry between marking and cutting or between cutting and ImageJ processing, which could have resulted in measurement inaccuracies. Moreover, photos of the cut-out leaflets were taken with a high-resolution hand-held camera, which can introduce image distortion. Photos were taken precisely planar with the target plane in an attempt at mitigating this error. Furthermore, a standard weakness of non-inferiority studies, compared with superiority studies, is that poor conduct of the trial or deviations from the protocol could result in false rejection of the null hypothesis that the experimental method is inferior. The calculated sample size required for a superiority study based on the pilot experiment was quite large (*n* = 356), therefore, a non-inferiority study was pursued. The goal of the study was not to determine superiority of the laser over the temple method, but, rather, to establish proof-of-concept for the use of laser projection in cardiovascular applications through comparison with a standard cardiac surgery technique.

## CONCLUSIONS

In summary, validation of a laser projection platform has demonstrated proof-of-concept of an alternative methodology for the preparation of surgical patches for use in congenital cardiac surgery. The favourable results of our validation study suggest that this laser-based system is appropriate for playing a key role in emerging methodologies for patient-specific reconstructive surgery involving cardiovascular patches.

## Data Availability

All relevant data are within the manuscript and its Supporting Information files.

## ABBREVIATIONS

AV: Aortic valve
CAD: Computer-aided design
FBP: Fixed bovine pericardium
OR: Operating room
PE: Percent error
PMPA: Porcine main pulmonary artery
PTFE: Polytetrafluoroethylene
SD: Standard deviation
UFBP: Unfixed bovine pericardium
